# Medicinal Cannabis (MedCan 3): a randomised, multicentre, double-blind, placebo-controlled trial to assess THC/CBD (1:20) to relieve symptom burden in patients with cancer—a study protocol for a randomised controlled trial

**DOI:** 10.1186/s13063-024-08091-z

**Published:** 2024-05-01

**Authors:** Taylan Gurgenci, Janet Hardy, Georgie Huggett, Karyn Foster, Anita Pelecanos, Ristan Greer, Jennifer Philip, Alison Haywood, Ruwani Mendis, Patsy Yates, Phillip Good

**Affiliations:** 1 Mater Research Institute, University of Queensland, Brisbane, Australia; 2 QIMR Berghofer Medical Research Institute, Herston, Australia; 3 Torus Research, Brisbane, Australia; 4 University of Melbourne, Melbourne, Australia; 5 Griffith University, Nathan, Australia; 6 Western Health, Melbourne, Australia; 7 Queensland University of Technology, Brisbane, Australia; 8 Department of Palliative Care, St Vincent’s Private Hospital Brisbane, Kangaroo Point, Australia; 9 Department of Supportive and Palliative Care, Mater Health, Brisbane, Australia

## Abstract

**Background:**

Distressing symptoms are common in advanced cancer. Medicinal cannabinoids are commonly prescribed for a variety of symptoms. There is little evidence to support their use for most indications in palliative care. This study aims to assess a 1:20 delta-9-tetrahydrocannabinol/cannabidiol (THC/CBD) cannabinoid preparation in the management of symptom distress in patients with advanced cancer undergoing palliative care.

**Methods and design:**

One hundred and fifty participants will be recruited across multiple sites in Queensland, Australia. A teletrial model will facilitate the recruitment of patients outside of major metropolitan areas. The study is a pragmatic, multicenter, randomised, placebo-controlled, two-arm trial of escalating doses of an oral 1:20 THC/CBD medicinal cannabinoid preparation (10 mg THC:200 mg CBD/mL). It will compare the efficacy and safety outcomes of a titrated dose range of 2.5 mg THC/50mgCBD to 30 mg THC/600 mg CBD per day against a placebo. There is a 2-week patient-determined titration phase, to reach a dose that achieves symptom relief or intolerable side effects, with a further 2 weeks of assessment on the final dose.

The primary objective is to assess the effect of escalating doses of a 1:20 THC/CBD medicinal cannabinoid preparation against placebo on change in total symptom distress score, with secondary objectives including establishing a patient-determined effective dose, the effect on sleep quality and overall quality of life. Some patients will be enrolled in a sub-study which will more rigorously evaluate the effect on sleep.

**Discussion:**

MedCan-3 is a high-quality, adequately powered, placebo-controlled trial which will help demonstrate the utility of a THC:CBD 1:20 oral medicinal cannabis product in reducing total symptom distress in this population. Secondary outcomes may lead to new hypotheses regarding medicinal cannabis’ role in particular symptoms or in particular cancers. The sleep sub-study will test the feasibility of using actigraphy and the Insomnia Severity Index (ISI) in this cohort. This will be the first large-scale palliative care randomised clinical trial to utilise the teletrial model in Australia. If successful, this will have significant implications for trial access for rural and remote patients in Australia and internationally.

**Trial registration:**

ANZCTR ACTRN12622000083796. Protocol number 001/20. Registered on 21 January 2022. Recruitment started on 8 August 2022.

**Supplementary Information:**

The online version contains supplementary material available at 10.1186/s13063-024-08091-z.

## Introduction

Patients with advanced cancer report many disabling symptoms. Traditionally, the efficacy of palliative interventions has been evaluated by measuring their effect on a specific symptom. For example, the intervention of oral haloperidol is said to be an efficacious palliative intervention as it provides a statistically significant and clinically relevant reduction in nausea.

Medicinal cannabis (MC) has not demonstrated consistent efficacy for any particular symptom in the palliative care of patients with advanced cancer. This has not reduced public support for its use. Consumers perceive the potential benefit as a holistic one that helps them ‘feel better’ overall, without specifically impacting on an individual symptom.

The value of aligning outcome measures with what consumers consider important is increasingly recognised. This is especially the case for MC research, as consumers often have strongly held, well-articulated preconceptions of how MC should be used [1].

There is little high-quality evidence for MC in palliative care generally. There are even fewer high-quality trials assessing the role of MC in reducing total symptom burden in these patients, rather than assessing individual symptoms, e.g. pain or nausea [2]. Moreover, which particular cannabinoid or cannabinoid combination works best for symptom management is still yet to be determined. Our team has recently completed a randomised clinical trial (RCT) of cannabidiol (CBD) as a single agent, showing no reduction in total symptom burden compared to placebo [3]. A subsequent RCT is assessing a 1:1 THC/CBD preparation [4]. The present study will clarify whether another THC/CBD combination, namely a 1:20 THC/CBD oral preparation, will reduce the total symptom burden in advanced cancer. Both clinicians and consumers seem to suspect a benefit to sleep symptoms, and so, we are deliberately assessing sleep symptoms in a separate sub-study to see whether there is a benefit in this particular patient population [5].

## Ethics

Research ethics approval has been obtained from all participating sites—Mater Misericordiae Ltd Human Research Ethics Committee, HREC/MML/81780, and St Vincent’s Health and Aged Care Human Research and Ethics Committee, HREC_22-02_PGOO. All participants give written informed consent, and the study is conducted in accordance with the Good Clinical Practice guidelines.

## Methods

### Aims and objectives

The objective is to determine whether medicinal cannabinoids have a role in reducing symptoms in patients with advanced cancer who are receiving palliative care. We hypothesise that participants in the MC arm will experience a greater reduction in symptom distress than those in the placebo arm.

The primary objective is to define whether self-titrated increasing doses of a 1:20 THC/CBD oral suspension formulation will reduce a patient’s total symptom distress score (TSDS) more than a placebo at day 14. TSDS is the sum of all components on the nine-item Edmonton Symptom Assessment Scale (ESAS) [6].

Secondary objectives include (a) ascertaining the median dose of 1:20 THC/CBD formulation achieved when patients are asked to titrate to effect or tolerance within a pre-specified range; (b) describing MC-induced change in individual symptom scores at days 7, 21 and 28; and (c) assessing the change in total physical and emotional scores, global impression of change, anxiety and depression and sleep quality.

#### Study design

This is a pragmatic, multicentre, randomised, placebo-controlled, two-arm parallel trial of escalating doses of oral THC/CBD: 10 mg:200 mg/mL oral solution. Participants will self-titrate the dose over the first 14 days using a pre-defined dosing schedule. They will cease self-titration at the maximum day 14 dose or at that dose which relieves symptoms or is limited by adverse effects. This dose will be continued for a further 2-week period for documentation of any benefits and adverse effects. This study design is based on previous RCTs successfully undertaken by this group [3, 4, 7].

#### Study setting

Participants will be recruited from sites within the Queensland Palliative Care Research Group (QPCRG) in metropolitan Brisbane. Additionally, we will be offering patients in more rural and remote areas the ability to participate by utilising a teletrial model. In this model, local research nurses employed by a government-funded teletrial network will provide on-the-ground support to health professionals based in Mater Hospital Brisbane. Potential participants outside metropolitan areas will be reviewed by an on-site teletrial research nurse in person. These nurses will perform all eligibility assessments on-site with remote supervision. If eligible, trial medications will be couriered to the patient. Similarly, actigraphs will be couriered to patients who wish to enrol in the sleep-related sub-study described below. It is anticipated that this will be predominately an outpatient study (although inpatients are still eligible), with sites having the ability to access this participant population from their established clinical service.

All participants will be given standard palliative care according to the local practice of the recruiting centre. They will continue all current medications including opioids, antiemetics, sedatives and specific anti-cancer therapy (including chemotherapy, immunotherapy and radiotherapy).

#### Study participants

Patients are eligible if they have the following:

- An advanced histologically proven cancer diagnosis (metastatic or locally advanced)
- Ongoing review by a palliative care team
- An ESAS total symptom distress score (TSDS) of ≥ 10/90 for cancer-related symptoms with at least one ESAS individual score ≥ 3/10
- A performance status of ≥ 30/100 as measured by the Australia-modified Karnofsky Scale (AKPS)
- A negative THC urine test at the eligibility assessment
- The ability to swallow oral medications
- Are aged ≥ 18 years, English-speaking (or have an interpreter available)

Where applicable, potential participants must:

- Submit a urine sample to exclude pregnancy (female patients of reproductive potential)
- Agree to avoid pregnancy for the duration of the trial and 3 months after ceasing the study medication (female patients of reproductive potential)
- Agree to avoid fathering a child for the duration of the trial and three months after ceasing the study medication (male patients)
- Agree to avoid sperm donation for the duration of the trial and 3 months after ceasing the study medication (male patients)
- Agree not to drive any motor vehicle for the duration of the trial

Potential participants will be excluded if they have:

- A history of hypersensitivity to any cannabinoid
- Unstable, untreated cardiovascular disease
- Severe hepatic impairment (total bilirubin ≥ 1.5 times the upper limit of the normal range), aspartate aminotransferase (AST) and alanine aminotransferase (ALT) ≥ 3.0 times the upper limit of the normal range (subjects with liver metastases excluded if AST and ALT of ≥ 5.0 times the upper limit of normal)
- Severe renal impairment (eGFR ≤ 20 mL/min/1.73 m.^2^)
- A history of psychiatric disorder (severe depression or anxiety, personality disorder, psychosis, schizophrenia and/or suicidal ideation)
- Cognitive impairment (St Louis University Mental Status Examination (SLUMS) ≤ 20/30)
- A known substance use disorder (Alcohol, Smoking and Substance Involvement Screening Test (ASSIST) examination scoring > 27) for any substance
- A history suggesting drug diversion
- Participated in a trial of a new clinical entity within the prior 28 days
- Treatment with a new specific anti-cancer agent (chemotherapy, targeted or hormonal therapy) or radiotherapy within the last 7 days
- A nut allergy
- A positive urine test for the presence of THC

### Consent process

During the consent process, potential participants and their approved carers will be provided with all pertinent information, including information regarding any associated sub-studies by the research staff. This information will be in the form of a participant information sheet (PICF) and will be supported by offers of verbal clarification of any additional queries. The purpose, expected procedures, participant requirements, benefits, risks, burdens and anticipated or possible adverse effects will be covered by this process. Participants will be explicitly reminded they cannot drive or operate heavy machinery nor take any non-trial cannabinoid products whilst taking the trial medication.

Potential participants will be given as much time and space as is necessary to formulate any questions and come to a decision. Any questions will be addressed. The consent form will be completed by a study team member in accordance with ethics committee requirements. The form will be signed and dated by the participant and investigator.

There will be separate consent forms for the RCT and the sleep sub-study. Patients will only be able to participate (optionally) in the sleep sub-study component if they consent to the main RCT.

### Randomisation

Eligible patients will be assigned in equal numbers to one of the two arms at random. Random number tables will be used for computer-generated randomisation schedules at an independent location by the staff not otherwise involved in the trial. A central registry will hold treatment allocations as determined by a permuted block randomisation schedule. Each site will have its own block randomisation schedule leading to even allocation to each treatment arm within each site. Actions to minimise the risk of bias are detailed in Additional file 1: Table S1.

Following fully informed written consent, the procedure will be as follows:

- The research pharmacy will be notified of a new participant.
- The investigator will complete a trial prescription with the participant’s study ID number and provide this to the trial pharmacy.
- Based on the randomisation schedule, the pharmacist will dispense the appropriate medication in a bottle labelled ‘THC:CBD 10 mg:200 mg/mL or placebo’.
- Participant ID, allocation number, date of request, preparation and dispensing will be recorded by the site pharmacist for each instance.

### Intervention

Trial medication and placebo oil formulation will be provided by Little Green Pharma Ltd. Little Green Pharma is supplying the medication free of charge but has no input into the study design nor execution. They will have no role in the analysis or publication of results.

The intervention arm (arm 1) is an oil formulation of THC/CBD 10 mg/200 mg/mL for oral administration (LG Pharma Pty Ltd.) to be delivered over the dose range of THC:CBD 2.5 mg/50 mg (0.25 mL once daily) to THC:CBD 30 mg/600 mg per day (1 mL three times daily). The placebo (arm 2) is a taste and appearance-matched oil formulation without the active ingredient though otherwise indistinguishable. Both arms will otherwise receive the usual palliative care. No part of usual palliative care is prohibited as part of the trial.

Both active and placebo arms will be provided one 70-mL bottle containing 50 mL of the oil solution. Participants will be instructed to store the bottles at room temperature and away from light. Bottles will have a child-resistant cap. Participants will be educated about how to administer medication via a supplied syringe and how to follow the dosing schedule. They will be instructed to use only those syringes supplied by investigators. Participants will record their dosing in the provided dosing schedule diary. Any product remaining at the conclusion of the trial will be returned to the research officer and destroyed by the local site pharmacy in accordance with pharmacy guidelines. Participants who are enrolled through the teletrial infrastructure will have their trial medicine sent to their local Queensland Health (government) facility for safekeeping until they retrieve it. They will use it in a similar way and then return it to the facility at the end of the trial.

#### Dose titration

Dose titration will be in accordance with the dosing schedule starting at 0.25 mL once daily. The dose increases every 2 days until the participant is satisfied with symptom improvement, experiences adverse events that are not acceptable or reaches the maximum dose (at the end of the 14-day dose-titration phase). Participants will be reviewed by phone or in person two to three times per week during this phase. If participants are reluctant to increase their dose because of adverse effects, they can consult with the research staff and maintain the current dose or reduce the dose or dosing frequency. All deviations from the dosing schedule will be recorded by the research staff. Detection of unexpected deviation is aided by comparing the volume remaining with the expected volume remaining for that time point. Exceeding the maximum dose as specified in the dosing schedule is not permitted.

Participants will be given the opportunity to remain on their chosen dose for an additional 14 days (a total of 28 days) to monitor ongoing efficacy and adverse events.

Participants consenting to the sleep study will be provided with an accelerometer to be worn nightly for the duration of the sub-study.

### Assessments

The research staff will contact all participants twice weekly for the first 2 weeks in addition to outpatient clinic reviews on days 0, 7, 14, 21 and 28, with outcome measures recorded at each of these time points. In addition to clinical documentation, symptom burden will be assessed using the Edmonton Symptom Assessment Scale (ESAS). Documentation of disease status (e.g. response or progression of disease) will be assessed at days 14 and 28.

A routine haematology and biochemistry screen including liver function tests will be taken at eligibility/baseline assessment. Blood for C-reactive protein (CRP) as a basic test of inflammation will be taken at baseline, day 14 and day 28. All consenting participants will have a urine test to confirm no recent use of THC-related products as a pre-screen. Female participants of child-bearing potential will have a urine test to exclude pregnancy. Additionally, patients will receive a phone call from a research nurse twice per week in the first fortnight, and then once per week for the second fortnight. Adverse events will be assessed at all of these interactions.

Participants in the optional sleep sub-study will be assessed using the Insomnia Severity Index (ISI) and Sleep Numerical Scale at days 0, 7 and 14. They will wear an accelerometer on their wrists for 14 days to measure circadian activity, sleep quality and quantity. Actigraphy has been found to be a validated, objective way to measure sleep outcomes [8].

Participants will be contacted at day 56 (+ 4 weeks post-last dose) to assess for adverse events as well as record post-trial cannabinoid use. Death will be noted for all participants up to the census point. This protocol has been written in accordance with the Standard Protocol Items: Recommendations for Interventional Trials (SPIRIT) (Table 1) [9]. A SPIRIT guideline checklist can be found in the supplementary material.

**Table 1:**
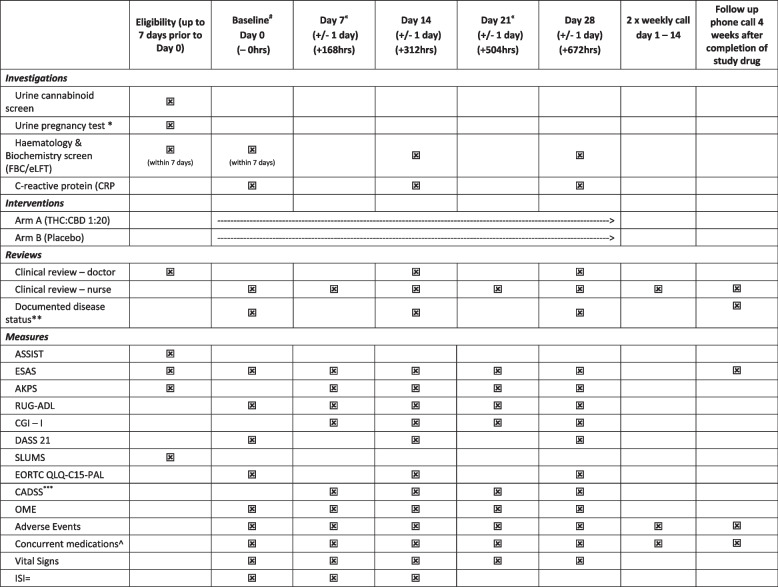
Study schedule

^*^Only those women of reproductive potential

^**^Confirm documented disease status

^****^CADSS only if indicated during adverse event assessment

^Including complementary medicine use

^#^Eligibility data to be used as a baseline as long as within 3 days of each other

^«^Day 7 and 21 review may be conducted via telehealth if deemed appropriate

^=^Optional sleep secondary aim 1 only—days 0–14

### Outcomes and assessment tools

The primary outcome is the change from the baseline of total ESAS TSDS at day 14. The ESAS is a nine-item inventory rated on an 11-point scale anchored at 0 (no problem) to 10 (worst imaginable of this problem). It assesses both physical and psychological symptoms, plus general well-being [6]. It has been validated in the assessment of symptoms in cancer patients [10].

Secondary outcomes include the following:

1. Patient-determined effective dose of THC/CBD 1:20 formulation, defined as the dose that achieves symptom relief with acceptable side effects.
2. ESAS TSDS at days 7, 21 and 28.
3. Physical and emotional ESAS scores at each time point.
  - Physical scores will be measured by the AKPS and Resource Utilisation Groups-Activities of Daily Living Scale (RUG-ADL). The AKPS is a validated variant of the Karnofsky Performance Status [11]. The Australian version can be applied to both inpatients and outpatients and is sensitive to changes in function over time. The RUG-ADL is an instrument developed for the measurement of nursing dependency [12]. The ADL scale measures patients’ needs for assistance in activities of daily living (eating, bed mobility, transferring and toileting).
4. Individual symptom scores (descriptive analysis only).
5. Oral morphine equivalent (OME), average use at baseline and weekly.
6. Patient Global Impression of Change (PGIC), days 7, 14, 21 and 28.
  - This is a subjective measure of symptom change completed by the participants themselves [13].
7. Clinical Global Impressions (CGI) scale, days 7, 14, 21 and 28.
  - This is a subjective measure of symptom change completed by the investigating clinician [13].
8. The Depression, Anxiety and Stress Scale (DASS-21).
  - DASS-21 is a self-reported 21-item scale, 7 questions per sub-item questionnaire measuring depression, anxiety and stress [14]. The DASS-21 will be used for assessment at baseline (day 0) and at days 14 and 28.
9. EORTC score QoL baseline and days 14 and 28.
  - This is a QoL measure found valid for use in a wide variety of cancer populations. To reduce patient burden, we will use the 15-question subset commonly used for the palliative care population [13].
10. NCI common terminology for adverse events V4.03, days 2, 4, 7, 9, 11, 14, 16, 18, 21, 23, 25 and 28.
  - The NCI CTCAE is a severity grading system for adverse events [15]. Adverse events relate to an unfavourable or unintended sign, symptom or disease temporarily associated with a medical treatment or a procedure which may not be related to the medical treatment or procedure. The CTCAE has a grading of 1 (mild) to 5 (death). The known common adverse events associated with cannabinoids are confusion, somnolence, paranoia, anxiety, mood changes, psychosis, hypertension, tachycardia, hyperhidrosis, nausea, vomiting and abdominal pain.
  The study will assess adverse events (AE) and serious adverse events (SAE) using the criteria of the NCI CTCAE V4.0. The known common AEs associated with THC/CBD will be sought at each time point. A comprehensive clinical review which includes the compilation of a concurrent medication list will occur at days 0, 7, 14, 21 and 28. This will include over-the-counter and complementary medications in addition to prescribed medications.
11. Sleep
  - ESAS (with additional sleep score item) at days 0, 7 and 14.
  - Objective measure of sleep via actigraphy. These measures will include total sleep time, sleep efficiency, wake after sleep onset and sleep onset latency (min). Participants will wear an accelerometer on their wrists for 14 days to measure circadian activity, sleep quality and quantity. Actigraphy has been found to be a validated, objective way to measure sleep outcomes [8].
  The ISI is a measurement tool that assesses sleep quality. It consists of 7 questions each of which are scored 0 (best) to 4 (worst) with respect to sleep quality [16]. Interpretation is by the total score which classifies 0–7 as no clinically significant insomnia, 8–14 as subthreshold insomnia, 15–21 as clinical insomnia of moderate severity and 22–28 as severe clinical insomnia. The Sleep Numerical Score is an 11-point numerical rating scale where 0 is the best possible sleep, and 10 is the worst possible sleep.

### Statistical analysis and sample size

We anticipate 150 participants will need to be randomised to achieve a sample size of 60 participants per arm to complete day 14. This allows for 20% attrition, consonant with the palliative care literature and previous RCTs. The sample size calculations are based on an effect size of ≥ 6 improvement in TSDS for the active arm compared to placebo, 80% power, 1:1 randomisation scheme, a type 1 error of 5% (two-tailed) and a standard deviation of 11.6. These numbers are based on the existing literature which suggests the minimum clinically important difference in the TSDS for a similar patient population to be 5.7 [6]. As such, we have elected to use an improvement of the TSDS of ≥ 6 as a clinically significant change. The Stata software (StataCorp. 2013. Stata Statistical Software: Release 13. College Station, TX: StataCorp) was used to estimate the sample size.

The study is powered to detect the superiority of arm 1 over arm 2 (placebo). Superiority will be determined by comparison at day 14 with appropriate baseline adjustment.

Participant demographic and clinical characteristics will be used to generate descriptive data and frequency distributions. For the primary outcome, the difference in change in TSDS between the treatment groups will be assessed using *t*-tests for unadjusted differences and ordinary least squares regression to adjust for baseline values [17]. Generalised estimating equations or mixed models will be used for secondary outcomes with multiple measurements per participant, accounting for within-subject correlation and effects due to the centre. Covariate influence will be explored.

An interim analysis will be completed following 50% completion of day 14. This analysis will be by an independent biostatistician. They will be blinded to treatment allocation. A report will be provided to the investigators and the Data Safety Monitoring Board (DSMB). This is to monitor participant safety. AEs and SAEs will be categorised by type and severity. The frequency of such adverse effects will be compared between the groups using the chi-squared tests and logistic regression to adjust for any relevant group differences.

If the interim analysis shows a significant difference, the investigators and the DSMB will be unblinded to the study groups and make any stopping decision on the basis of the nature of any AEs and/or SAEs and ethical grounds, as well as consideration of any statistical differences between the groups. Grounds for stopping on evidence of clear benefit will be considered. The Peto approach will be taken with symmetric stopping boundaries at *p* = 0.001.

A detailed statistical analysis plan will be prepared and ratified by the DSMB.

The sleep part of the study will be powered to detect a mean difference of ≥ 6 on the ISI between the intervention arm and the placebo group. Assuming a 1:1 ratio of active and placebo treatment, a mean difference between the groups (reduction in ISI score) of ≥ 6, a standard deviation of the difference of 8, alpha of 0.05 and power of 80%, 58 participants, 29 in each group, would be needed. To allow for 20% attrition, 74 subjects (37 in each group) will be recruited.

All protocol modifications will be approved by the relevant Human Research Ethics Committee and communicated to all investigators, and trial registration information updated. All investigators will have access to the final dataset. The datasets used and/or analysed during the study will be available from the corresponding author upon reasonable request.

### Data collection and management

The study is expected to be based mostly in the outpatient clinic, though inpatients are eligible. Most data will be collected and then recoded in the appropriate CRF or questionnaire. A small amount of data will be taken from the medical record. Most data will be collected as described in Table 2.

**Table 2:** Data collection plan

Measure	Source	Collected by
General medical information	Clinical record	Medical officer
General demographic data	Clinical record	Study nurse
Pathology results—blood	Pathology report	Pathology
Pathology results—urine	Clinical record	Study nurse
Vital signs	CRF/clinical record	Study nurse
Concurrent medications/OME	Clinical record	Study nurse
Study trial data—questionnaires—ASSIST, SLUMS, EORTC, QLQ-C15-PAL	CRF	Patient, study nurse, medical officer
Study trial data—ESAS, AKPS, RUG-ADL, PGIC, CGI-S/l, DASS-21	CRF	Patient, study nurse, medical officer
Side effects—safety	CRF, clinical record	Study nurse, medical officer

All data collected will be kept in a patient file (identified by ID number only). All data will be stored in a locked filing cabinet. All CRFs will be stored at study completion. Electronic files will be password-protected. The files themselves will be stored within a locked office. Hospital policy regarding medical records will be followed at all times.

The trial will be conducted with permission and in accordance with Queensland and Australian Department of Health regulations, on the use of medicinal cannabis and subject to approval and monitoring by each clinical site’s HREC. An independent DSMB to include an independent statistician, palliative care specialist, medical experts, researchers and consumers will be formed and will meet regularly, with primary responsibility for monitoring adverse and serious adverse events. All AEs and SAEs will be reviewed at a minimum of 6 monthly intervals, or more frequently if needed.

### Post-trial care

There is no anticipated harm or compensation for trial participation. Participants may be able to access ongoing cannabinoid products through entry into other open-label studies, but this is not guaranteed. This will be offered to rural and remote patients through a telehealth clinic. The manufacturer may offer a reduced price based on need at their discretion but this is not promised. All clinical investigators will be approved authorised cannabis prescribers and will be able to continue to prescribe, as long as the participant can fund their own supply. Participants will be informed of approved products and suppliers. The dose and formulation used post-study will be at the discretion of the patient and prescriber.

### Dissemination

A lay summary of the results will be offered to participants and carers. A media release will be generated for the popular press as well as for local and national trade newsletters (CPCRE, PCNA, PCQ, ANZSPM, CNSA, CareSearch, PaCCSC). There will be a social media release as well as presentations to local consumer groups and end-users, including but not limited to patient education evenings, wellness/survivorship groups, Mater Foundation and local charity groups. Researchers will present at local, national and international scientific meetings and inform relevant peak bodies and societies (ANZSPM, COSA, PCA). There is no intention to use professional writers to help with peer-reviewed publications.

## Discussion

This study is the third in a series of three placebo-controlled, randomised trials by these investigators to assess the efficacy of MC in patients with advanced cancer. The use of the total symptom burden as the primary outcome is a major strength of this study as it is more consistent with how consumers report the purported benefits of medicinal cannabis in this context. The sleep sub-study will help test the common consumer belief that medicinal cannabis helps with sleep disorders in cancer [1].

Placebo randomisation is as important in palliative medicine as other specialties and this study includes detailed procedures to maintain blinding. This will avoid biassing treatment effect estimation. The use of a titration schedule rather than a fixed dose is pragmatic and, again, consistent with how consumers use medicinal cannabis in this setting. This will improve the generalisability of the result. The study will provide valuable objective data on the dosing of this specific THC/CBD formulation.

This trial would be the first double-blinded, placebo-controlled trial in Australian palliative medicine to use a teletrial model. Successful recruitment through this model would support greater trial participation for rural and remote palliative patients, who currently have extremely limited access to clinical trials in this specialty. This trial therefore raises an important mechanism to improve equity in the care of people with advanced illness.

## Trial status

ANZCTR: ACTRN12622000083796. Protocol number 001/20. Registered on 21/1/2022. Recruitment started on 8/8/2022. The estimated date of the last patient recruitment is 02/05/2025. Estimated date of the last data collection is 30/05/2025. Status: recruiting.

### Supplementary Information

**Additional file 1: Table S1.** Bias minimization measures.

**Additional file 2. **

## References

[1] Lintzeris N, Mills L, Suraev A, Bravo M, Arkell T, Arnold JC (2020). Medical cannabis use in the Australian community following introduction of legal access: the 2018–2019 Online Cross-Sectional Cannabis as Medicine Survey (CAMS-18). Harm Reduct J.

[2] Doppen M, Kung S, Maijers I, John M, Dunphy H, Townsley H (2022). Cannabis in palliative care: a systematic review of current evidence. J Pain Symptom Manage.

[3] Hardy J, Greer R, Huggett G, Kearney A, Gurgenci T, Good P (2023). Phase IIb randomized, placebo-controlled, dose-escalating, double-blind study of cannabidiol oil for the relief of symptoms in advanced cancer (MedCan1-CBD). J Clin Oncol.

[4] Hardy J, Haywood A, Gogna G, Martin J, Yates P, Greer R (2020). Oral medicinal cannabinoids to relieve symptom burden in the palliative care of patients with advanced cancer: a double-blind, placebo-controlled, randomised clinical trial of efficacy and safety of 1:1 delta-9-tetrahydrocannabinol (THC) and cannabidiol (CBD). Trials.

[5] Arkell TR, Downey LA, Hayley AC, Roth S (2023). Assessment of medical cannabis and health-related quality of life. JAMA Netw Open.

[6] Hui D, Shamieh O, Paiva CE, Khamash O, Perez-Cruz PE, Kwon JH (2016). Minimal clinically important difference in the physical, emotional, and total symptom distress scores of the Edmonton Symptom Assessment System. J Pain Symptom Manage.

[7] Good PD, Greer RM, Huggett GE, Hardy JR (2020). An open-label pilot study testing the feasibility of assessing total symptom burden in trials of cannabinoid medications in palliative care. J Palliat Med.

[8] Berger AM, Wielgus KK, Young-McCaughan S, Fischer P, Farr L, Lee KA (2008). Methodological challenges when using actigraphy in research. J Pain Symptom Manage.

[9] Chan AW, Tetzlaff JM, Altman DG, Laupacis A, Gotzsche PC, Krleza-Jeric K (2013). SPIRIT 2013 statement: defining standard protocol items for clinical trials. Ann Intern Med.

[10] Hui D, Bruera E (2017). The Edmonton Symptom Assessment System 25 years later: past, present, and future developments. J Pain Symptom Manage.

[11] Abernethy AP, Shelby-James T, Fazekas BS, Woods D, Currow DC (2005). The Australia-modified Karnofsky Performance Status (AKPS) scale: a revised scale for contemporary palliative care clinical practice [ISRCTN81117481]. BMC Palliat Care.

[12] Williams BC, Fries BE, Foley WJ, Schneider D, Gavazzi M (1994). Activities of daily living and costs in nursing homes. Health Care Financ Rev.

[13] Davis MP, Hui D (2017). Quality of life in palliative care. Expert Rev Qual Life Cancer Care.

[14] Fox RS, Lillis TA, Gerhart J, Hoerger M, Duberstein P (2018). Multiple group confirmatory factor analysis of the DASS-21 Depression and Anxiety Scales: how do they perform in a cancer sample?. Psychol Rep.

[15] US Department of Health Human Services (2009). Common Terminology Criteria for Adverse Events (CTCAE) version 4.0.

[16] Schulte T, Hofmeister D, Mehnert-Theuerkauf A, Hartung T, Hinz A (2021). Assessment of sleep problems with the Insomnia Severity Index (ISI) and the sleep item of the Patient Health Questionnaire (PHQ-9) in cancer patients. Support Care Cancer.

[17] Vickers AJ, Altman DG (2001). Statistics notes: analysing controlled trials with baseline and follow up measurements. BMJ.

