# Correction: Medicinal Cannabis (MedCan 3): a randomised, multicentre, double-blind, placebo-controlled trial to assess THC/CBD (1:20) to relieve symptom burden in patients with cancer—a study protocol for a randomised controlled trial

**DOI:** 10.1186/s13063-024-08195-6

**Published:** 2024-05-30

**Authors:** Taylan Gurgenci, Janet Hardy, Georgie Huggett, Karyn Foster, Anita Pelecanos, Ristan Greer, Jennifer Philip, Alison Haywood, Ruwani Mendis, Patsy Yates, Phillip Good

**Affiliations:** 1 Mater Research Institute, University of Queensland, Brisbane, Australia; 2 QIMR Berghofer Medical Research Institute, Herston, Australia; 3 Torus Research, Brisbane, Australia; 4 Department of Medicine, Western Health, Melbourne, Australia; 5 Grifth University, Nathan, Australia; 6 Queensland University of Technology, Brisbane, Australia; 7 Department of Palliative Care, St Vincent’s Private Hospital Brisbane, Kangaroo Point, Australia; 8 Department of Supportive and Palliative Care, Mater Health, Brisbane, Australia

**Correction** **:** **Trials 25, 293 (2024)**

**https://doi.org/10.1186/s13063-024-08091-z**

Following the publication of the original article [1], we have been notified that Jennifer Phillip’s affiliation should read as 'University of Melbourne' instead of 'Western Health'.

The original article has been corrected.

## References

[1] Gurgenci (2024). Medicinal Cannabis (MedCan 3): a randomised, multicentre, double-blind, placebo-controlled trial to assess THC/CBD (1:20) to relieve symptom burden in patients with cancer—a study protocol for a randomised controlled trial. Trials.

