# Supplementary material for: Medicinal Cannabis (MedCan 3): a randomised, multicentre, double-blind, placebo-controlled trial to assess THC/CBD (1:20) to relieve symptom burden in patients with cancer—a study protocol for a randomised controlled trial
Source: Trials. 2024 May 1;25:293. doi: 10.1186/s13063-024-08091-z (PMC11064296; doi:10.1186/s13063-024-08091-z)
Supplement: Supplementary file 1 — Additional file 1: Table S1. Bias minimization measures. [file 13063_2024_8091_MOESM1_ESM.docx]

| **Supplementary Table 1 – Bias minimization measures** | |
| --- | --- |
| Bias arising from the randomisation process | Schedules for treatment allocation were developed for each site using permuted blocks with randomly allocated block sizes. These were computer generated at an independent centre by a statistician not involved in the analysis.  Random assignment schedules will be held by the central registry and provided to the study pharmacists. Trial pharmacists, who have no clinical or regulatory responsibilities associated with the conduct of the study, will dispense active or inactive medication for the participant according to the schedule. The participant ID, allocation number, date of request, preparation, and dispensing will be recorded in a log maintained by the site pharmacist for each random assignment. The randomisation schedule and all pharmacy source documents that could link a treatment number to a treatment assignment will be secured (locked in pharmacy with limited access) until study completion.  In case of the need for emergency unblinding, the allocation schedule will be sent under password protection to two associates at external sites who have no role in the running of the clinical trial.  All participants, caregivers, investigators, and clinical staff will remain blind to study assignment until completion of the trial and data analysis. All study drugs and placebo oil solutions will be identical in appearance and matched for taste, color and bottle size and supplied in identical dark glass bottles. Labelling of the bottles will not provide information as to which arm had been allocated. |
| Bias due to deviations from intended interventions | A consort diagram will be presented. Primary analysis will be per-protocol. However, an intention-to-treat analysis will also be performed and presented as part of the additional results. |
| Bias in measurement of the outcome | All participants, caregivers, investigators, research assistants and clinical staff will remain blind to study assignment until completion of the trial and data analysis.  The biostatistician will be blinded as to treatment group for analysis of the primary outcome. |
| Bias due to missing outcome data | The attrition rate at 2 weeks (time of primary outcome measure) is expected to be approximately 20%. The study avoids excessive patient-completed forms to reduce the likelihood of missing outcome data. The reasons for drop-out will be clearly presented in a Consort diagram. |
| Bias in selection of the reported result | All planned outcome measures will be reported. If they are not, this deviation will be made explicit. |
| Bias arising from participant selection | Baseline demographics and relevant variables will be clearly summarised and presented in a table. Selection bias will be reduced due to allocation concealment and randomisation as previously described. |
